# Vaginal Myomectomy for Large Intracervical Fibroids in Women Desirous of Preserving Their Uterus: A Case Series

**DOI:** 10.7759/cureus.82788

**Published:** 2025-04-22

**Authors:** Neeru Malik, Meenakshi Sidhar, Sandhya Jain, Neha Agrawal, Nikita Madan

**Affiliations:** 1 Obstetrics and Gynaecology, Dr. Baba Saheb Ambedkar Medical College and Hospital, New Delhi, IND; 2 Pathology, Dr. Baba Saheb Ambedkar Medical College and Hospital, New Delhi, IND; 3 Obstetrics and Gynaecology, Employees State Insurance - Postgraduate Institute of Medical Sciences and Research (ESI-PGIMSR), New Delhi, IND

**Keywords:** bonney’s principles, cervical fibroid, leiomyomas, myomas, vaginal myomectomy

## Abstract

Cervical fibroids are rare, benign tumors. The treatment of uterine fibroids is well-established, with standard treatment guidelines in place; however, there remains a lack of consensus on a standardized approach for cervical fibroids.

A small prolapsing fibroid polyp may be removed vaginally, and a hysteroscope can be used in such cases to identify and ligate the pedicle. However, large cervical fibroids present a surgical challenge. Myomectomy is the cornerstone for the surgical management of cervical fibroids in women who wish to preserve their uterus. An enlarged cervix alters the anatomy of adjacent vital structures like the ureters, bladder, rectum, and uterine vessels, increasing the risk of injury to these structures. Due to the narrow operating field and the potential for injury, preoperative catheterization of the ureters through double-J (DJ) stenting is performed to delineate their course prior to surgery. Here, we report a series of cases in which intracervical fibroids, visible vaginally as a cervical protuberance, were enucleated vaginally using Bonney's principles. The base of the fibroid was clamped and ligated, followed by obliteration of the space and reconstruction of the cervix. Injection of vasopressin was unavailable in our resource-limited hospital; therefore, adrenaline was injected in a 1:200,000 dilution into the fibroid capsule intraoperatively to minimize blood loss. This method of enucleation minimizes the risk of injury to adjacent organs since the dissection occurs intracapsularly. Preoperative prophylactic DJ stenting was also not needed.

Despite the lack of advanced medical options to reduce fibroid size and vascularity, such as preoperative gonadotropin-releasing hormone (GnRH) analogues and uterine artery embolization, in our low-resource setting, our technique of vaginal myomectomy effectively managed the challenges presented by large intracervical fibroids while preserving fertility. The only limitation was that fibroids that were not palpable through the cervical lips could not be enucleated using this approach.

## Introduction

Uterine fibroids (myomas or leiomyomas) are the most common benign tumours in women of reproductive age, affecting 70% of women globally [1]. Of the total cases of fibroids, only less than 0.6% are cervical fibroids [2]. The majority of female patients undergoing surgery for symptomatic fibroids are between 31 and 50 years of age [3]. Fibroids develop from smooth muscle fibres with concentric spiral patterns and fibrous connective tissue, which tends to be anchored to the myometrium by fibromuscular bridges forming a pseudocapsule [4]. Fibroids are mostly asymptomatic, but they are also a significant source of clinical morbidity [5]. Symptomatic fibroids have a clinical presentation of abnormal uterine bleeding, pressure symptoms, pain, infertility, and obstetric complications [5]. Pressure effects on the bladder or urethra and dystocia are caused by cervical fibroid [2].

Fibroid is an oestrogen and progesterone-dependent tumour; therefore, it is rare before menarche and its growth regresses after menopause [4]. Various risk factors for fibroids include early menarche, late menopause, nulliparity, family history of uterine fibroids, and high body mass index (BMI) [6]. Despite the introduction of new nonsurgical treatment techniques like uterine artery embolization and myomectomy, hysterectomy remains the mainstay for the treatment of fibroids. A conservative surgical approach, such as vaginal myomectomy or laparoscopic myomectomy, is an excellent option for patients who desire to preserve their fertility [7].

Cervical myomas are divided into two types, namely subserosal (extracervical type: anterior, posterior, or lateral) and submucosal (intracervical type), which occur within the cervix [4]. Depending on their position, cervical fibroids can be anterior, posterior, central, or lateral. The cervix is surrounded by important pelvic structures, such as the ureters, urinary bladder, and rectum, which are at a high risk of injury as their anatomy may be altered by ballooning of the cervix due to the presence of the fibroid. Significant haemorrhage can occur if the nearby uterine arteries are injured. A narrow operative field and difficult repair of the big cavity render the procedure technically challenging. There is, at present, no consensus on the ideal surgical approach for intracervical fibroids.

In this case series, we share our experience of a novel technique of vaginal myomectomy of large intracervical leiomyomas extending into the body of the uterus in young women desirous of preserving their fertility. 

## Case presentation

In this case series, we have included five cases of women with large intracervical fibroids desirous to preserve their uterus who underwent successful vaginal myomectomy at Dr. Baba Saheb Ambedkar Medical College and Hospital, New Delhi, India. All five cases were done under spinal anaesthesia. The patients were informed of the advantages and risks involved, following which written informed consent was obtained. The patients whose fibroid was accessible from the cervical lip were taken for this study.

Case 1

**Figure 1:**
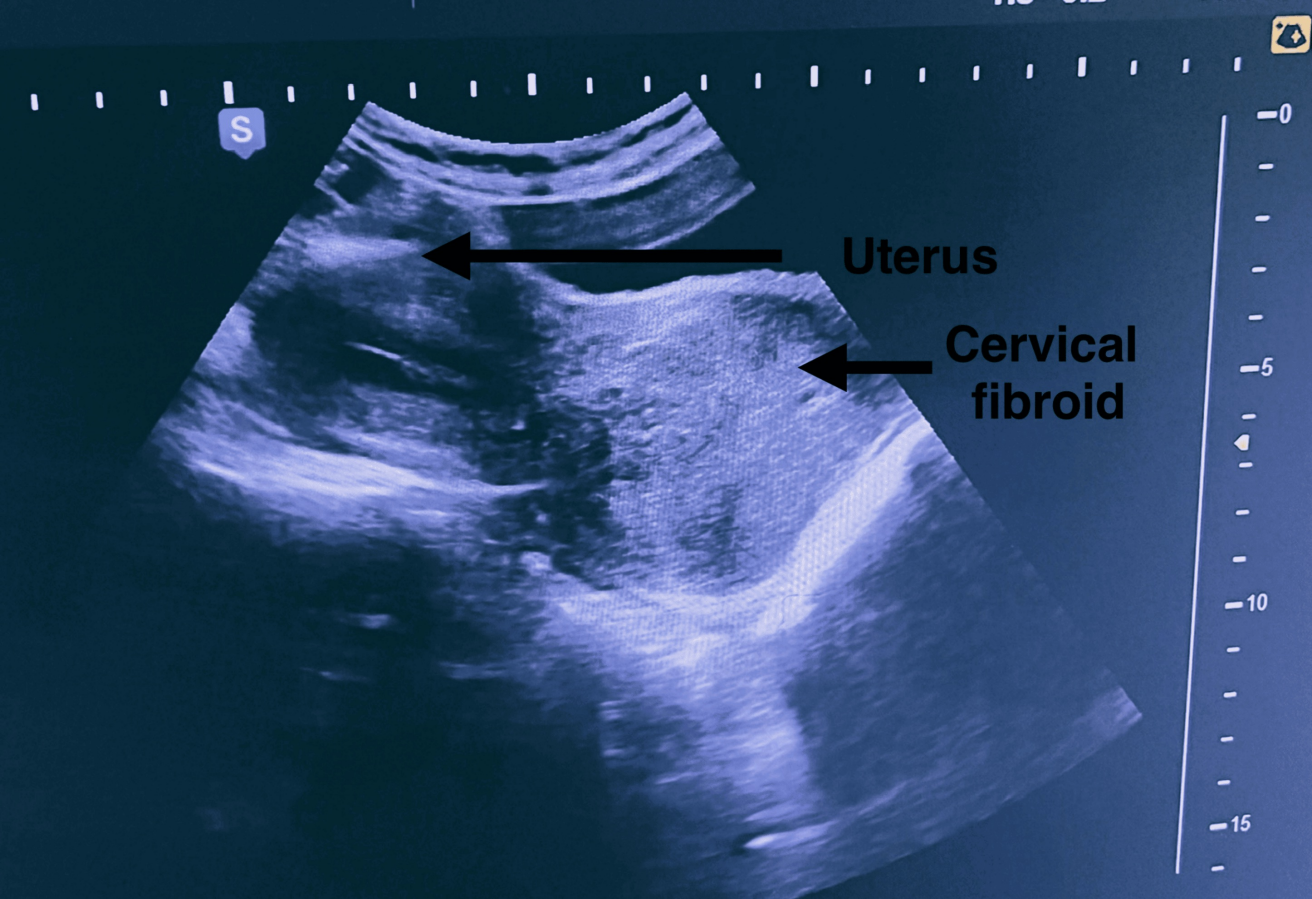
Ultrasound image showed a well-defined heteroechoic lesion measuring 6.7 x 7.4 x 7.2 cm (around 180 cc) in the lower uterine body and cervix, likely suggestive of a large cervical fibroid

A P1L1 (para 1, living 1) woman, 35 years old, having a previous normal delivery, presented to our hospital with a history of polymenorrhagia. She did not have any pressure symptoms. The abdomen was soft and non-tender, and no abdomino-pelvic mass was felt. On per speculum examination, the anterior lip of the cervix appeared bulky. The posterior lip of the cervix was thin and spread over the mass. On bimanual pelvic examination, an 8 x 7 cm mass was felt in the anterior cervix extending upwards into the body of the uterus. The upper margin could not be felt. Ultrasonography revealed the uterus was anteverted with 7.5 mm endometrial thickness. A well-defined heteroechoic lesion that measured 6.7 x 7.4 x 7.2 cm (around 180 cc) in the lower uterine body and cervix was noted, likely suggestive of a fibroid, as shown in Figure 1.

**Figure 2:**
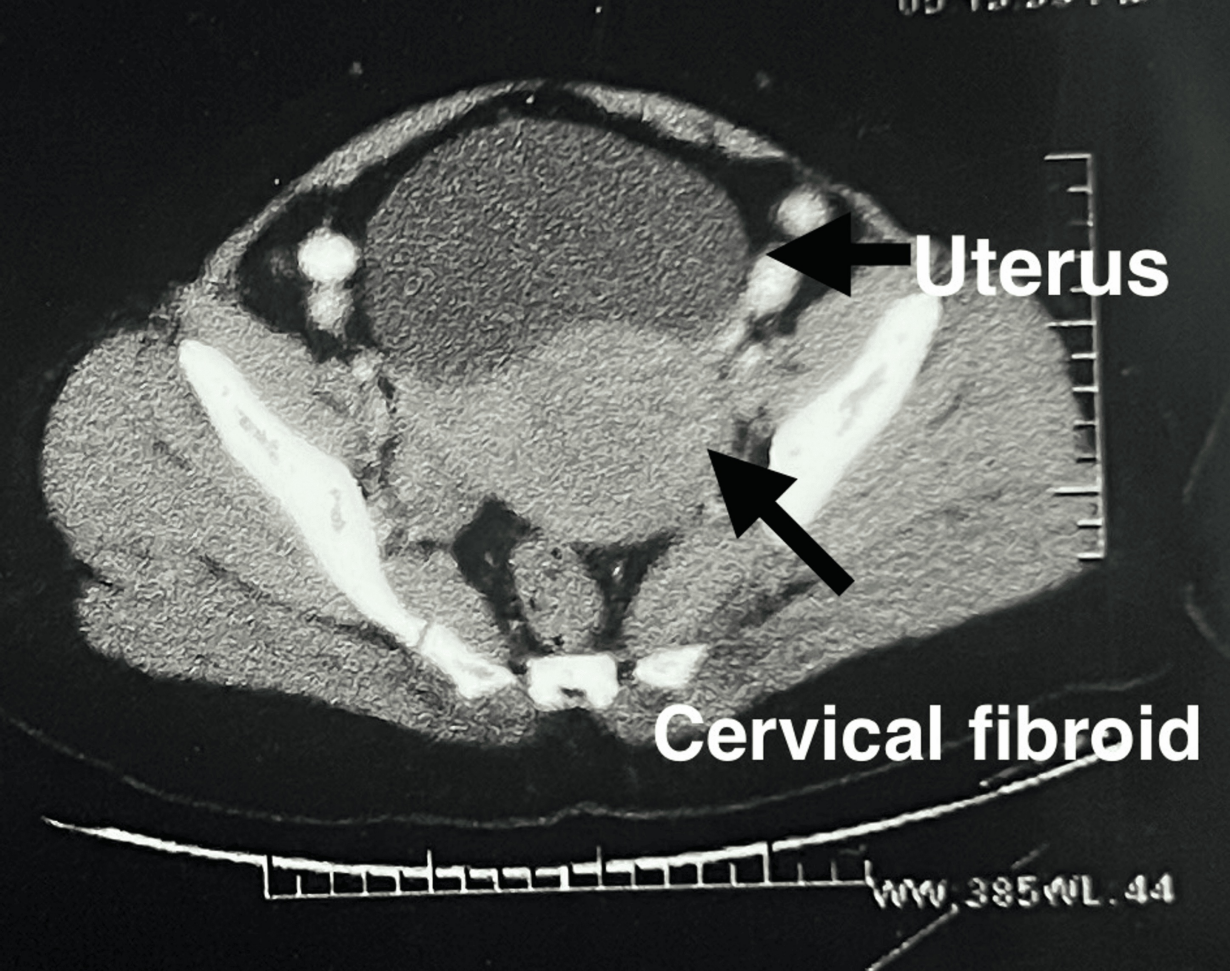
CT of the abdomen and pelvis with contrast revealed a cervical fibroid in the left lateral wall extending till the cervical region of the uterus, measuring 8.4 x 7.3 cm

CT abdomen and pelvis with contrast revealed a large fibroid in the left lateral wall extending till the cervical region of the uterus, measuring 8.4 x 7.3 cm, with bilateral polycystic ovaries, as shown in Figure 2.

Operative Steps

**Figure 3:**
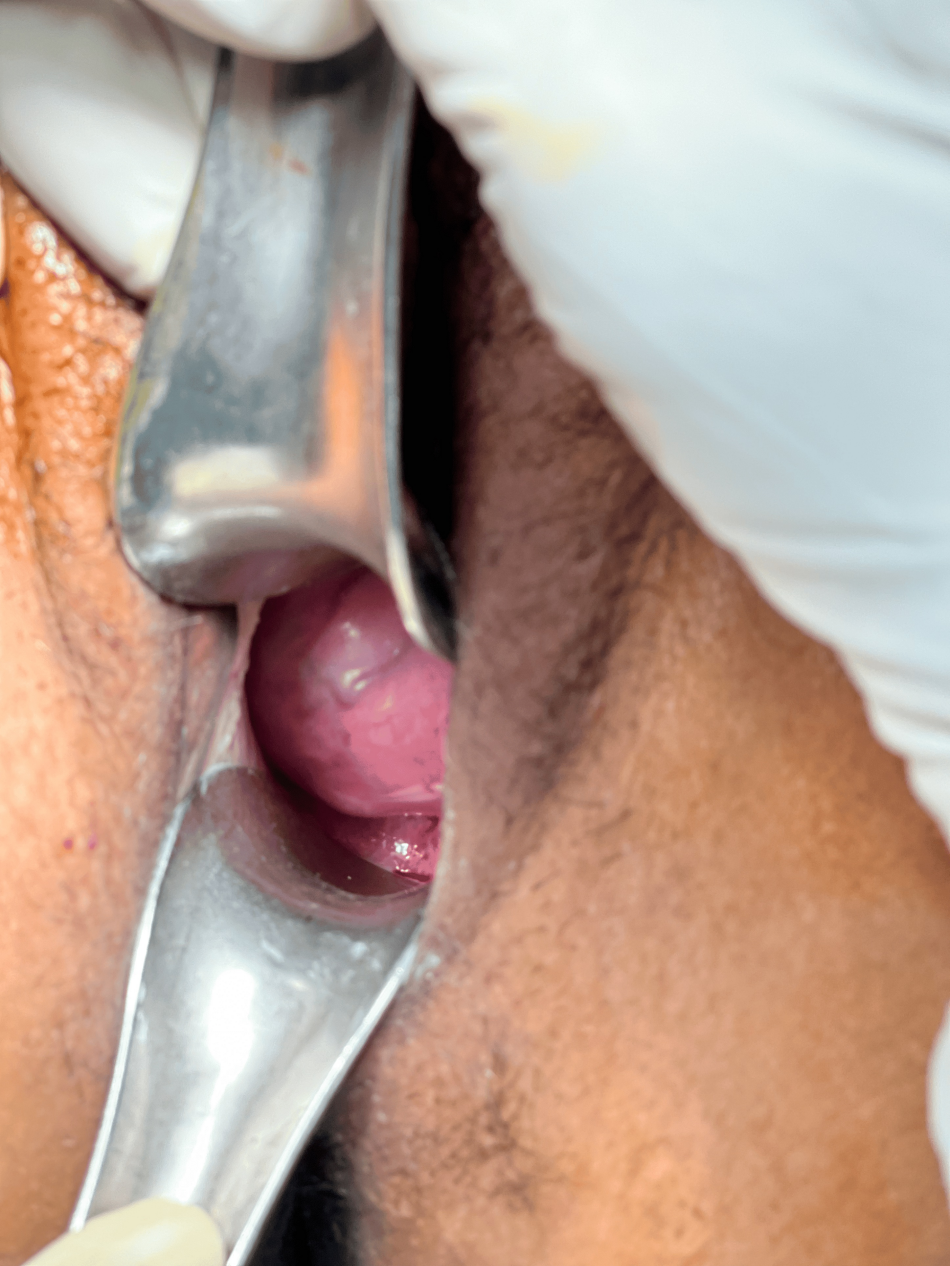
The bulky anterior lip of cervix showing increased vascularity and protuberance due to underlying cervical fibroid

**Figure 4:**
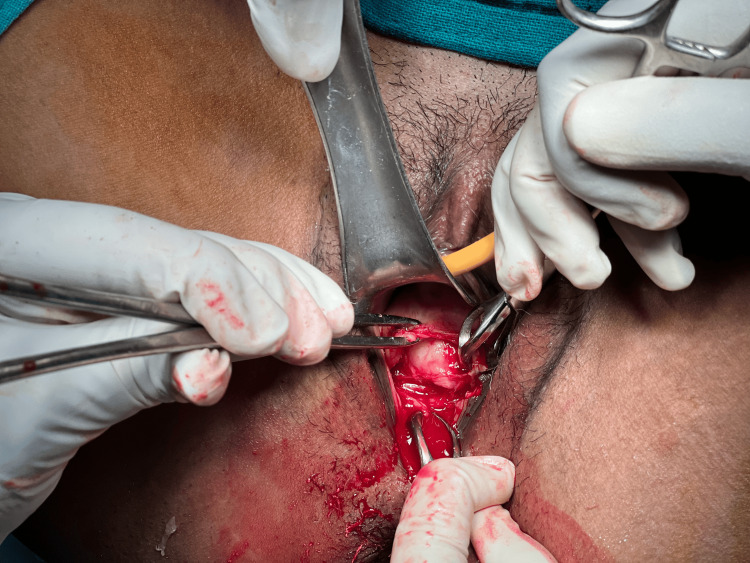
A tranverse incision was given on the bulky anterior lip of the cervix following Bonney's principles

**Figure 5:**
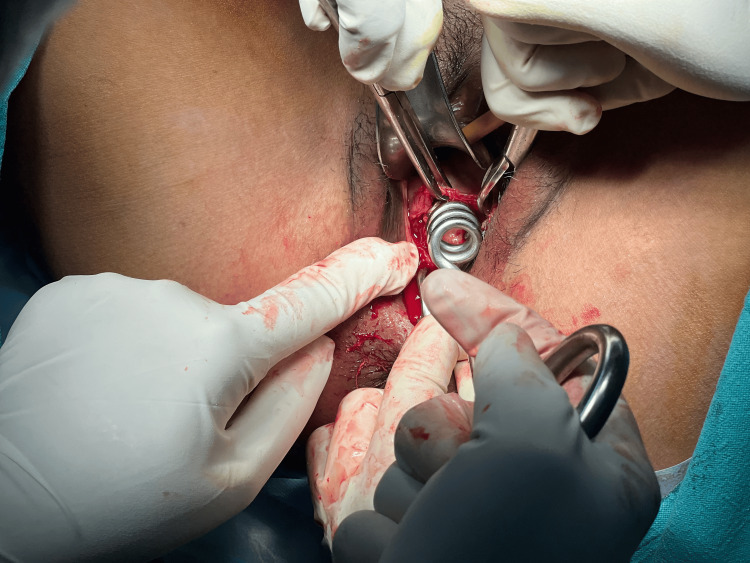
A myoma screw was inserted into the substance of fibroid

**Figure 6:**
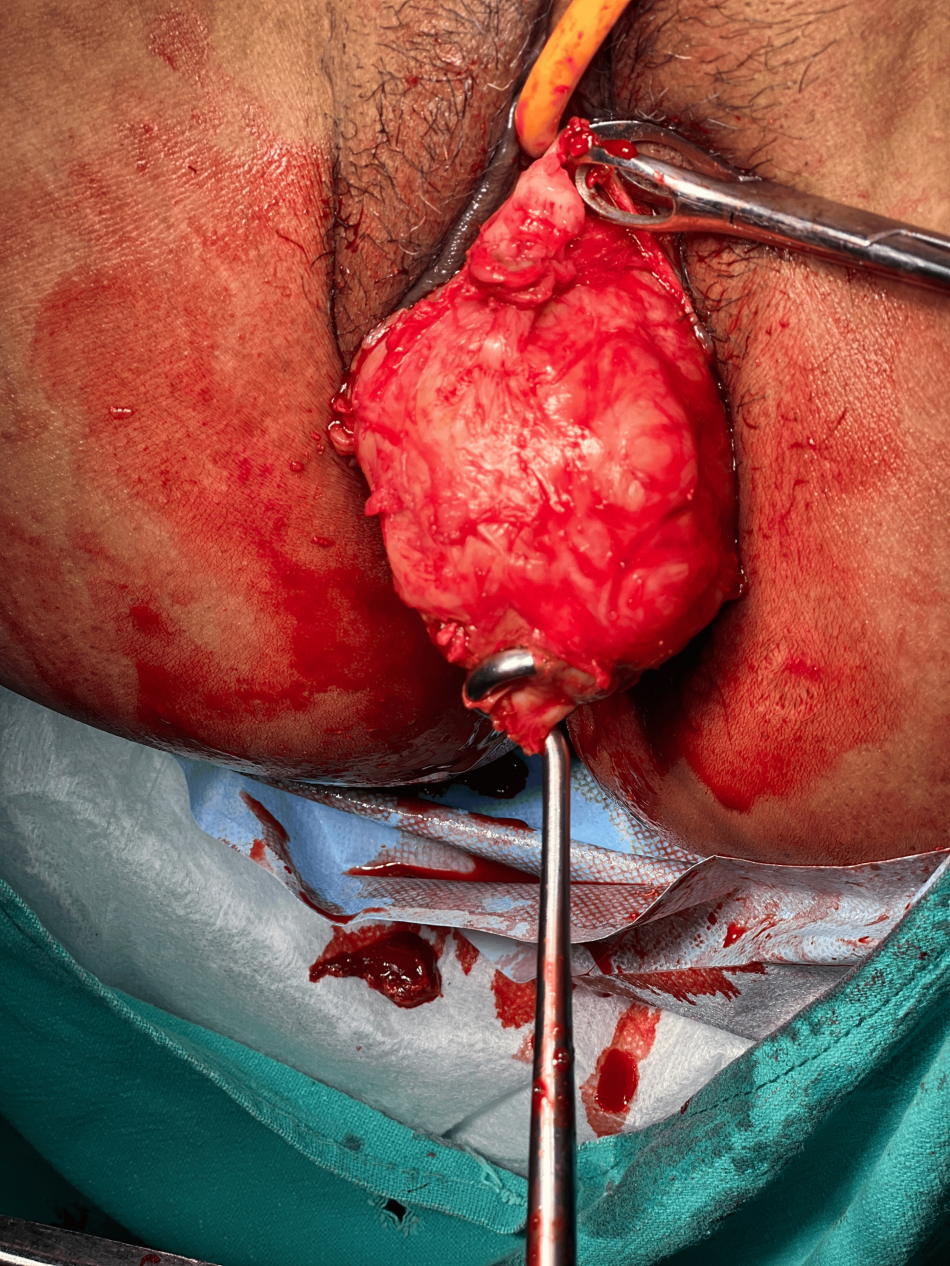
Fibroid being removed vaginally after enucleation

**Figure 7:**
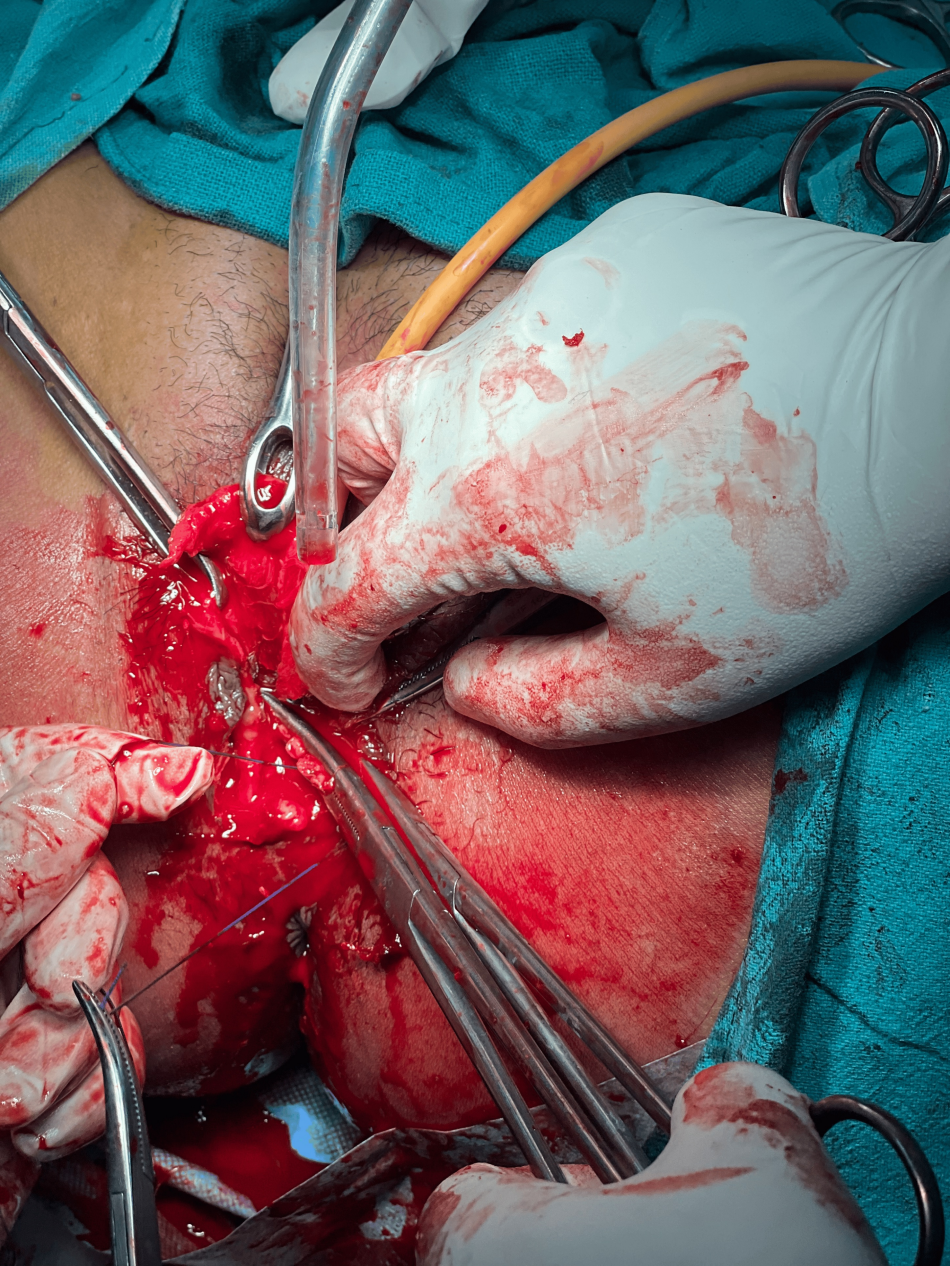
The base of the fibroid containing feeding vessels was clamped and ligated

The patient consent was taken for vaginal myomectomy. She was also counselled for a high possibility of a hysterectomy. The patient lay in a dorsal lithotomy position, and under all aseptic precautions, an examination under anaesthesia was performed. Fibroid was noted to be arising from the anterior lip of the cervix, as shown in Figure 3, and going upwards into the body of the uterus with a right lateral extension into the broad ligament. The posterior lip of the cervix was held with Allis tissue holding forceps and pulled downwards. Vaginal mucosa and fibroid capsule were injected with 1:200,000 adrenaline saline solution. A transverse incision was given over the fibroid anteriorly, as shown in Figure 4. The fibroid was held with a myoma screw (Figure 5). After ascertaining the right plane of cleavage, the fibroid was enucleated with blunt and sharp dissection (Figure 6). The tumour was gradually enucleated down to its base by using the fingers through the plane of cleavage. Further, the vascular base of the fibroid was clamped and ligated (Figure 7).

**Figure 8:**
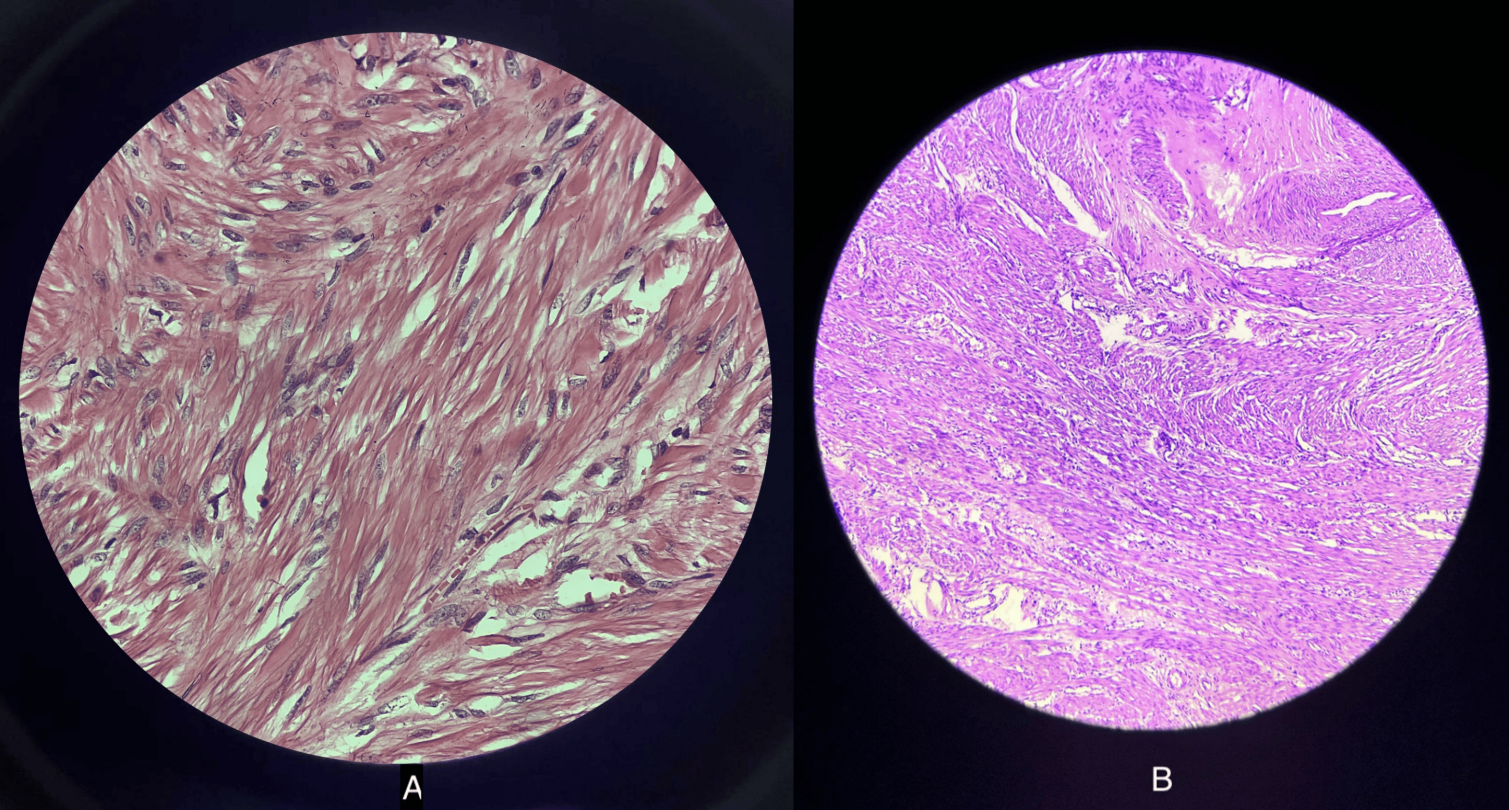
High power magnification 40x (A) and low power magnification 10x (B) of the cervical fibroid

The cavity was obliterated by applying the mattress suture with polyglactin no. 1. Redundant tissue was removed, and haemostasis was secured. The anterior lip of the cervix was reconstructed. The cervix was dilated. Endometrial curetting was obtained and sent for histopathological examination. Vaginal packing was done. Average blood loss was around 150 to 200 mL. After 24 hours, the vaginal pack was removed, and the patient was discharged on postoperative day 4. Postoperatively, the patient was counselled regarding complications in subsequent pregnancy and advised to avoid pregnancy for at least one year. Histopathological examination of the fibroid confirmed it as a benign leiomyoma. Microscopically, it consisted of whorled anastomosing fascicles of uniform, spindle-shaped, smooth muscle cells with indistinct borders and eosinophilic cytoplasm. The nuclei were elongated with finely dispersed chromatin. There was no cytological atypia or necrosis. Occasional mitoses were seen. Blood vessels with thickened walls were also noted. The histopathological findings in high (40x) and low (10x) power magnification are shown in Figure 8.

The patient was relieved of her symptoms. She was followed up with six-monthly ultrasounds. Her follow-up over four years did not show recurrence of fibroid or cervical stenosis, as she had normal menstrual cycles.

Case 2 

**Figure 9:**
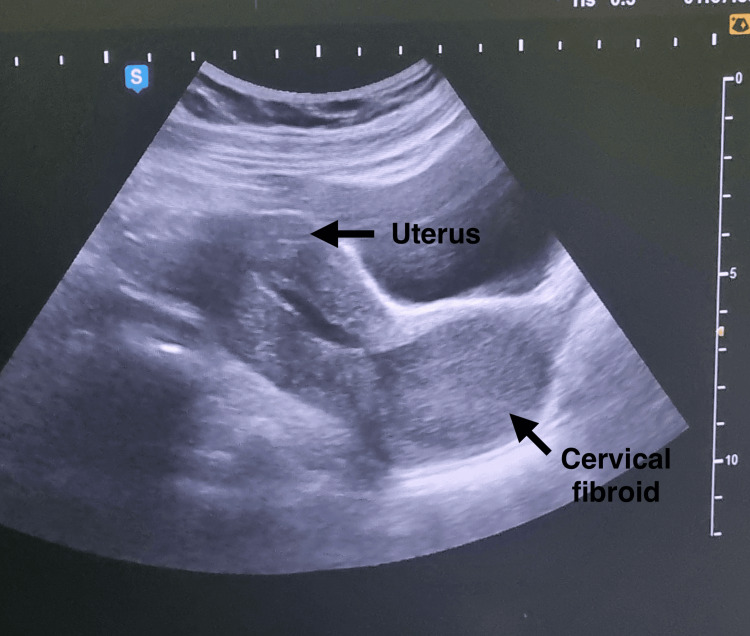
Ultrasound image showed a uterus of normal size with a well-defined heterogeneous lesion in the lower uterus and cervix, measuring 5.3 x 6.3 x 5.6 cm, provisionally diagnosed as a cervical fibroid

A P0L0 (para 0 living 0) woman, 32 years old, presented to our hospital with a history of abnormal uterine bleeding with dull aching pain in the abdomen for six months. Her medical and surgical history was uneventful. The abdomen was soft and non-tender, with no palpable abdomino-pelvic mass. On speculum examination, the anterior lip of the cervix appeared bulky with increased vascularity. A bimanual pelvic examination revealed a mass of 6 x 7 cm in the anterior cervix extending upwards into the body of the uterus. The upper margin could not be felt. Ultrasonography revealed a normally sized uterus anteverted with an endometrial thickness of 7.5 mm. A well-defined heterogeneous lesion was seen in the lower uterus and cervix, measuring 5.3 x 6.3 x 5.6 cm, with significant vascularity; provisionally (?) cervical fibroid (?) carcinoma (Figure 9). Bilateral adnexa was normal, with no free fluid noted.

**Figure 10:**
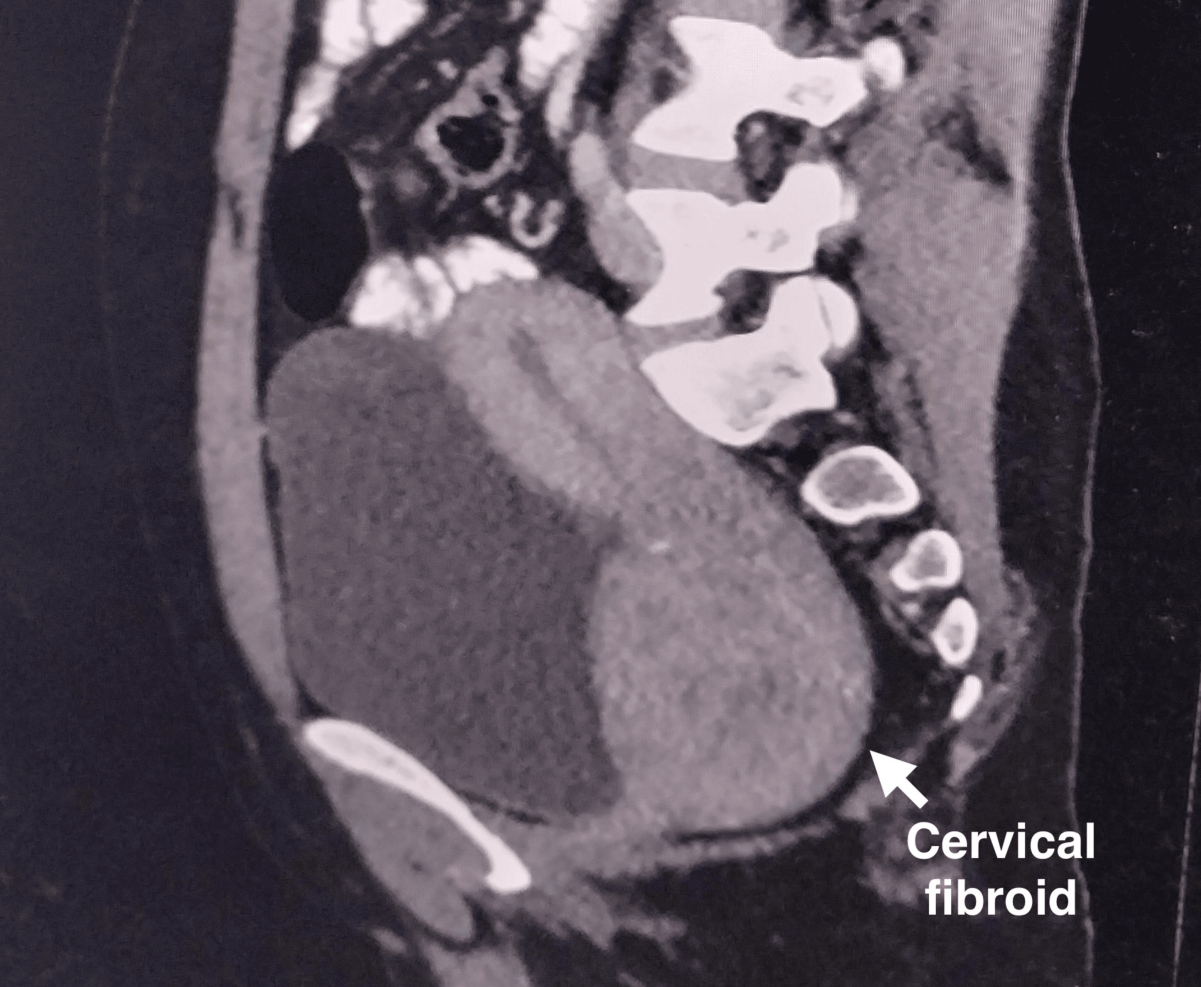
Contrast-enhanced CT revealed a heterogeneously enhancing, whorled appearing, iso to hypo-dense lesion measuring 5 x 6.3 x 6.3 cm in the endocervical region, likely representing a cervical fibroid replacing the anterior lip of cervix

Contrast-enhanced CT (CECT) revealed a heterogeneously enhancing, whorled appearing, iso to hypo-dense lesion measuring 5 x 6.3 x 6.3 cm in the endocervical region, likely a cervical fibroid replacing the anterior lip of the cervix, as shown in Figure 10.

**Figure 11:**
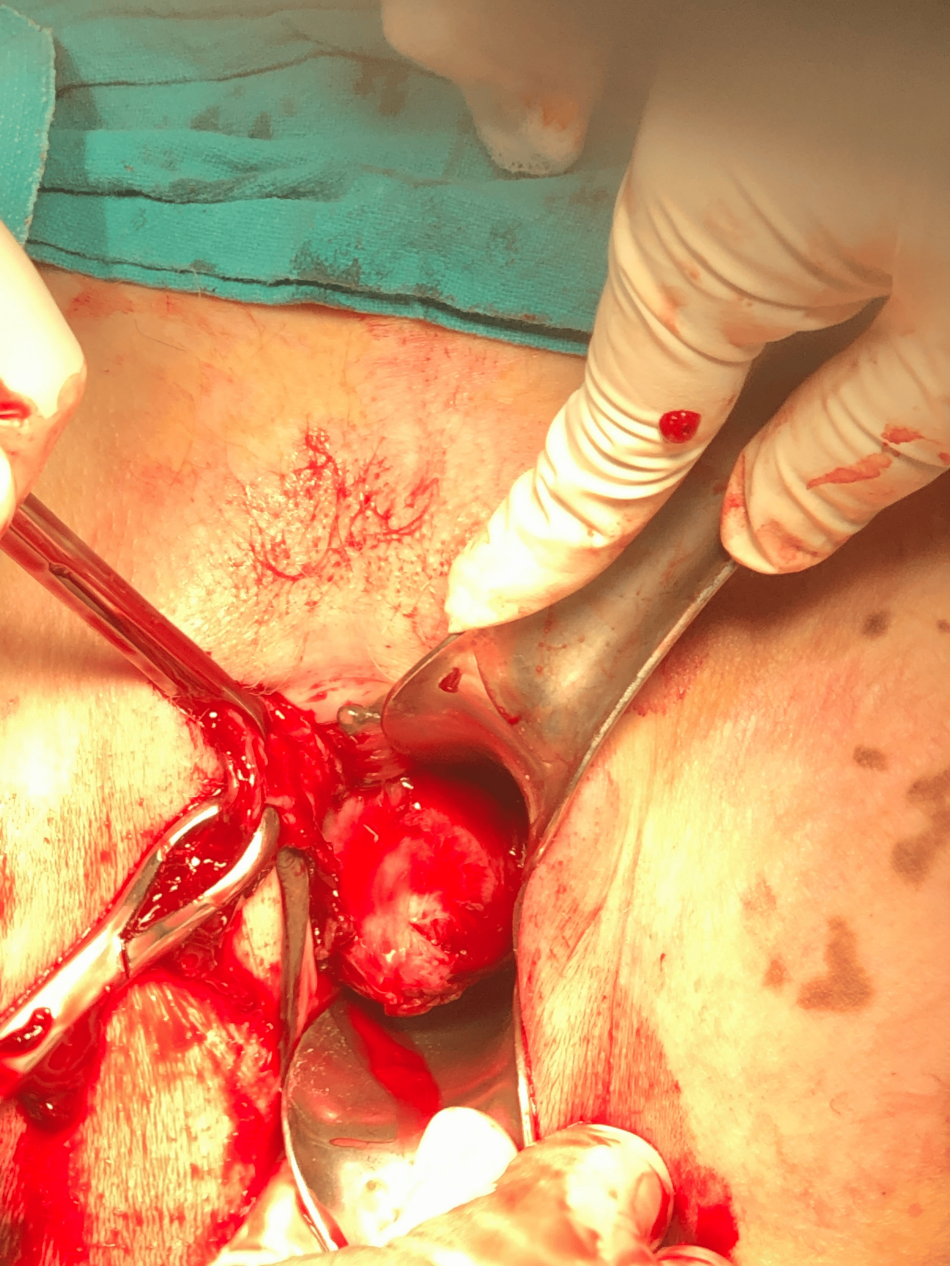
Vaginal myomectomy

The patient underwent a successful vaginal myomectomy using the steps outlined earlier (Figure 11). 

Histopathological examination confirmed a benign cervical leiomyoma. Her symptoms were relieved. She conceived spontaneously after a year and delivered a healthy baby by normal vaginal delivery. Upon follow-up, the patient did not develop cervical incompetence as her pregnancy had continued till term.

Case 3

A 35-year-old woman presented to the emergency department with complaints of menorrhagia and generalised weakness for one month and a history of excessive bleeding per vagina for last two days. She also had complaints of frequent micturition and dull aching lower abdominal pain for the last three months. However, she reported no history of any gynaecological malignancy in the family. Abdominal examination was unremarkable, and no ascites were noted.

On per speculum examination, a 12 x 10 cm large irregular mass was found arising from the anterior lip of the cervix. The posterior lip was thinned out. On per vaginal examination, 12 x 10 cm polypoidal growth involving the anterior lip of the cervix and going into the body of the uterus was noted with no pedicle. The uterus was bulky. The uterine sound was not able to pass into the cavity.

**Figure 12:**
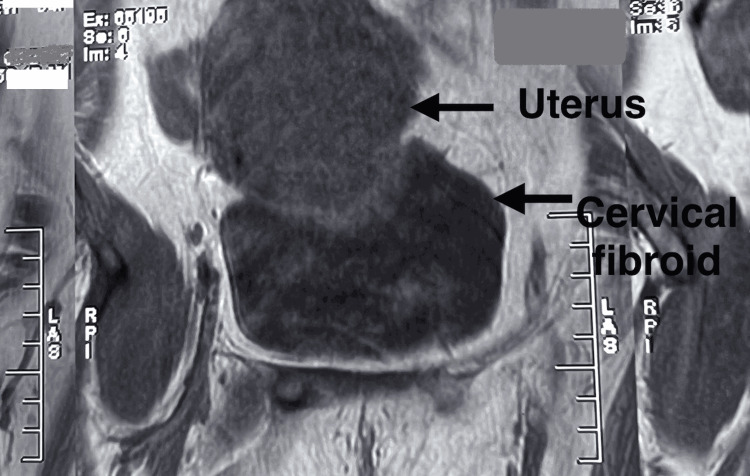
MRI revealed a uterus measuring 6.3 x 7.6 cm, with a large heterogeneous mass measuring 10 x 10 cm located at the lower uterine segment, suggestive of a cervical fibroid

On general examination, the patient was found to be very pale. Her lab investigation showed haemoglobin levels to be 4.1 g/dL, pulse rate 110/min, and systolic blood pressure (BP) 86 mmHg. MRI revealed uterine size of 6.3 x 7.6 cm, with a large heterogenous mass of 10 x 10 cm in the lower uterine segment, suggestive of a fibroid, as shown in Figure 12. Bilateral adnexa within normal limits were noted.

**Figure 13:**
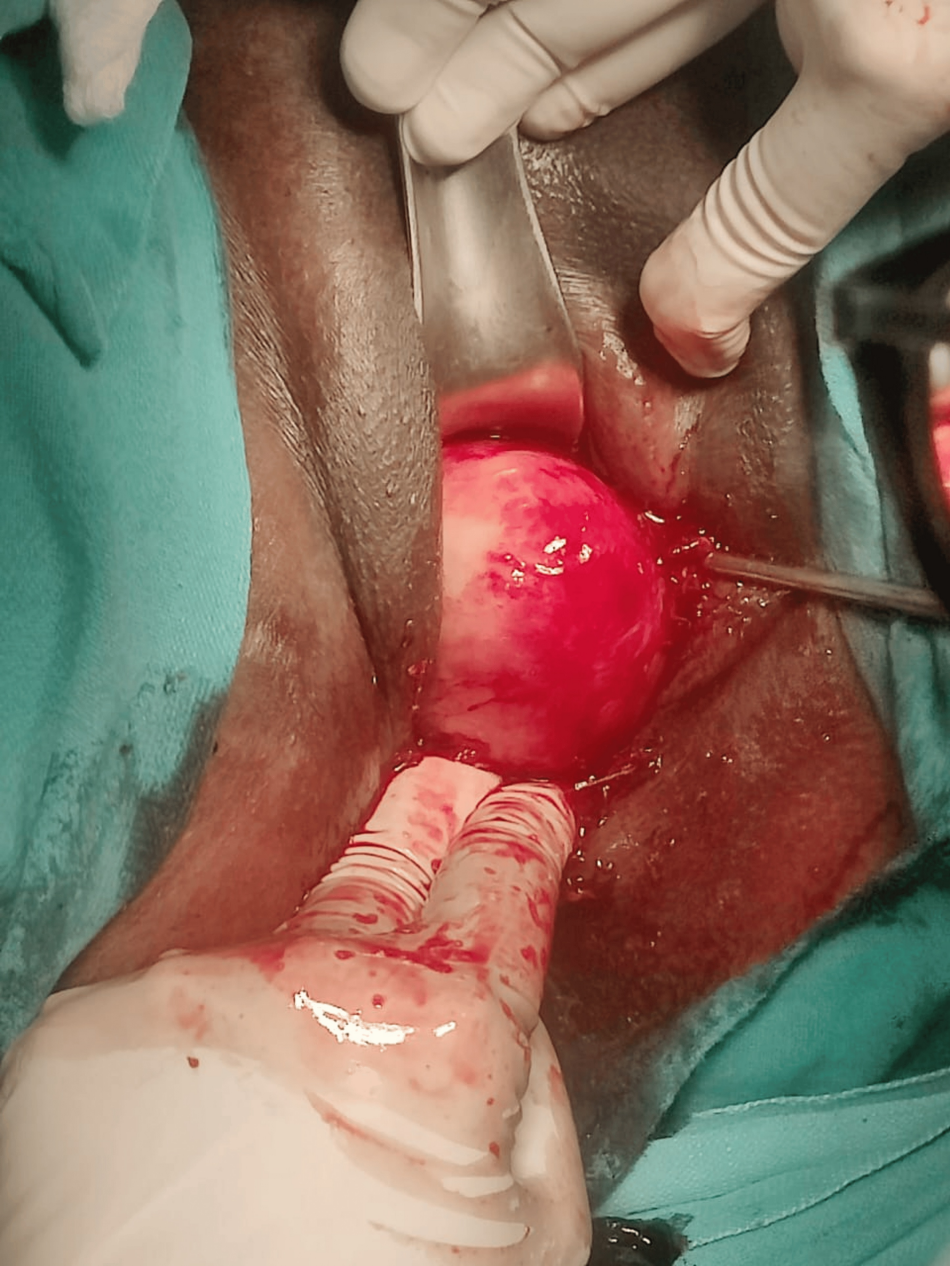
Fibroid being removed vaginally

As the patient was bleeding profusely, she was immediately transfused two units of blood and taken up for myomectomy as a semi-emergency case, and the fibroid was successfully enucleated vaginally (Figure 13). 

Histopathological examination (HPE) confirmed our diagnosis of benign cervical leiomyoma. Her menorrhagia was relieved. Her follow-up ultrasound did not show recurrence of cervical fibroid, and the patient had resumed normal menstruation.

Case 4 

A P2L2 (para 2, living 2) woman, 36 years old, visited the outpatient department (OPD) with complaints of lower abdominal pain and dyspareunia for the past six months. She also had complaints of menorrhagia for the last four months. Her per abdominal examination was unremarkable. On per speculum examination, the anterior fornix was obliterated, and the cervix could not be differentiated from the mass. Bimanual examination revealed a mass of 6 x 6 x 6 cm occupying the anterior and right lateral fornix. The uterus felt bulky and enlarged.

**Figure 14:**
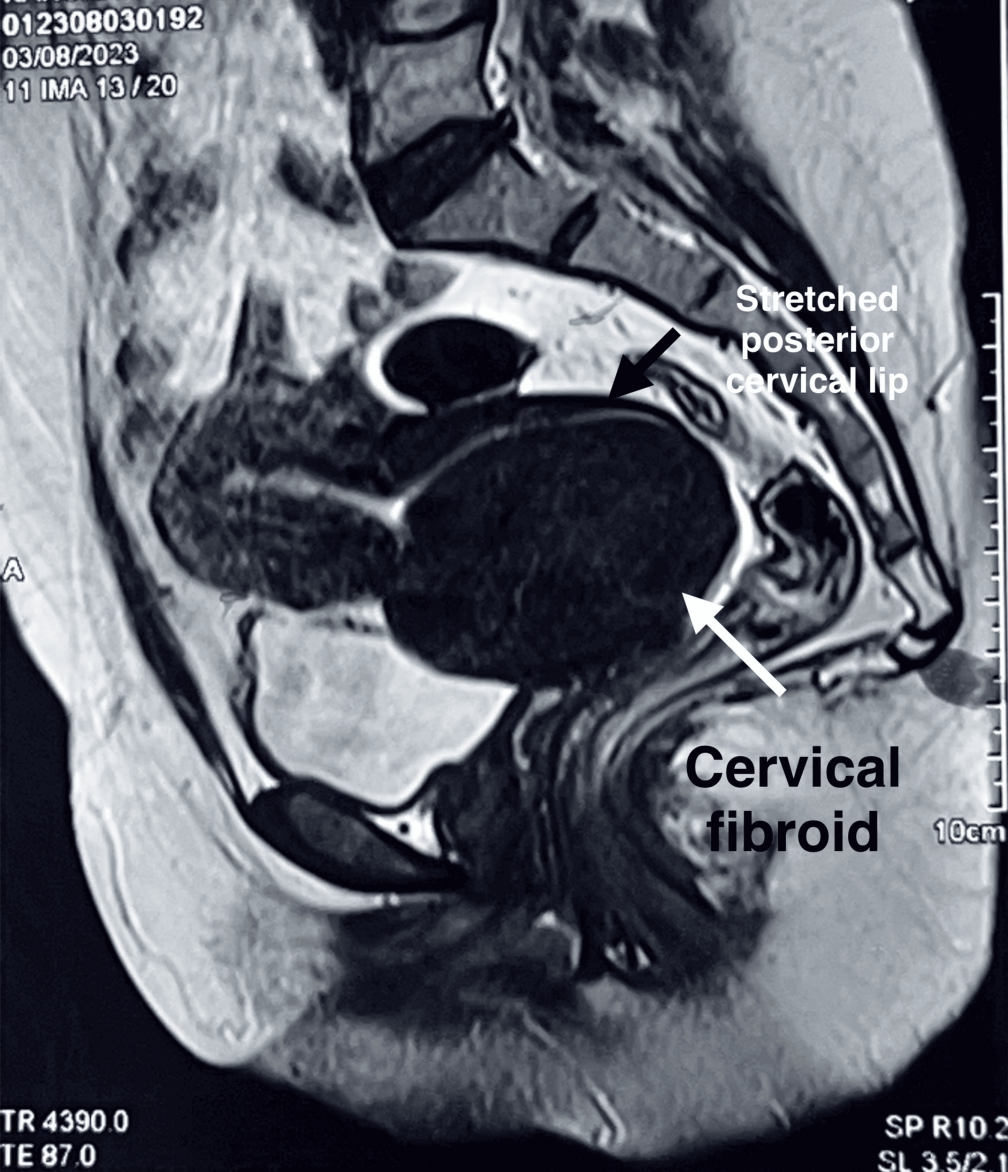
MRI was suggestive of a fibroid measuring 52 x 65 x 68 mm, involving the anterior lip of the cervix, with the posterior lip spread over it and extending into the vagina

 MRI suggested an anterior cervical fibroid of 52 x 65 x 68 mm (Figure 14).

**Figure 15:**
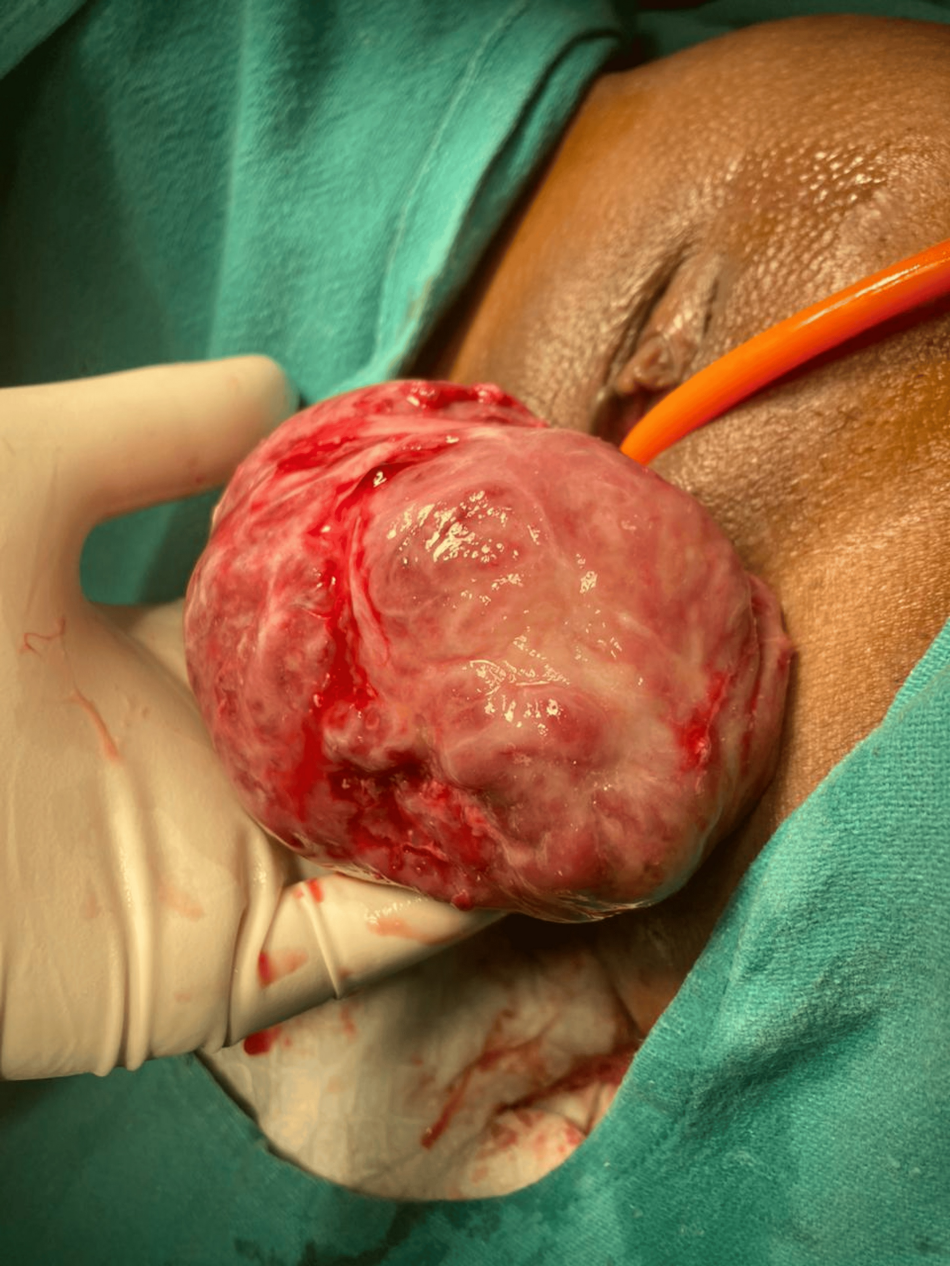
Large cervical fibroid replacing anterior lip of cervix removed by vaginal myomectomy

The lesion was compressing and displacing the cervical canal posteriorly. Endometrial thickness was 8 mm. Bilateral ovaries were normal. The patient was taken up for a vaginal myomectomy following the steps outlined above (Figure 15). There were no intraoperative or postoperative complications. Benign cervical leiomyoma was confirmed upon HPE. The patient made a complete and fast recovery. Her follow-up over the next three years showed normal menstruation and no dyspareunia.

Case 5

A P1L0 (para 1, living 0) woman, 30 years old, visited the OPD with complaints of intermenstrual bleeding for the last five months. She had no complaints of heavy menstrual bleeding or pressure symptoms.

**Figure 16:**
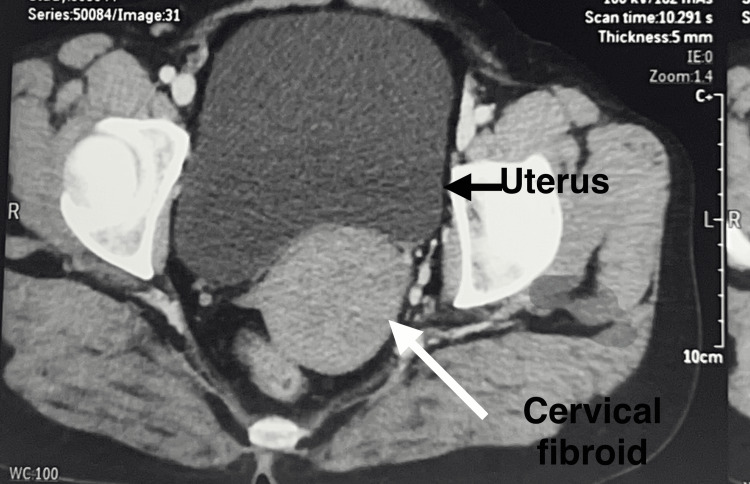
Contrast-enhanced CT of the abdomen and pelvis revealed a cervical fibroid measuring 6 x 7 cm

On abdominal examination, no abdominal mass was palpable. On per speculum examination, a sessile cervical fibroid arising from the anterior lip of the cervix, 6 cm in size, was noted. Vaginal examination revealed a bulging cervix containing a large mass, with the external cervical ostium (os) displaced posteriorly. On bimanual examination, the uterus was bulky in size. No adnexal mass was felt. CECT confirmed the presence of a large cervical leiomyoma of size 6 x 7 cm (Figure 16).

**Figure 17:**
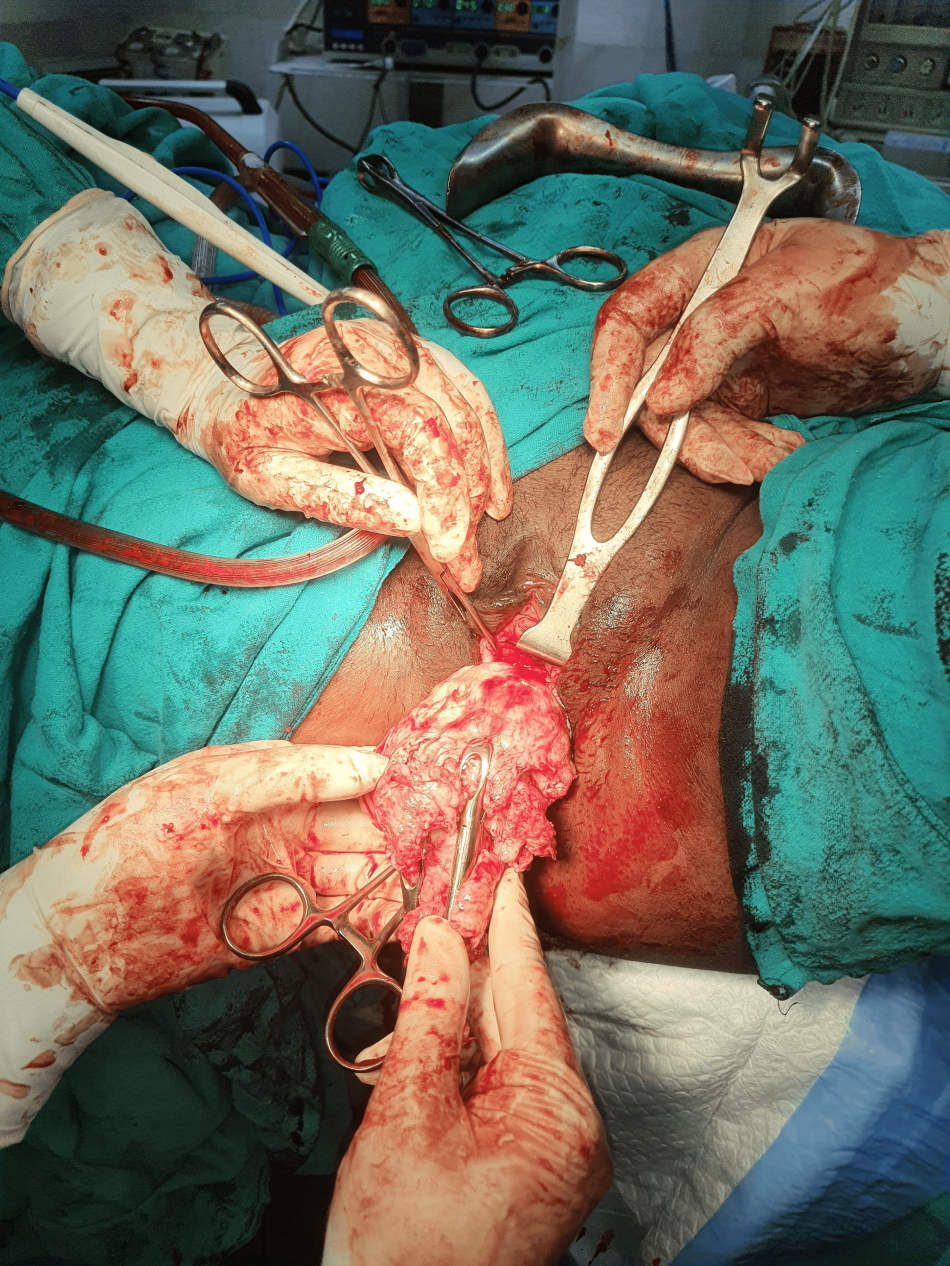
Cervical leiomyoma being removed vaginally

Vaginal myomectomy was done following the steps outlined above, and the uterus was preserved (Figure 17). The patient made a fast recovery. The patient conceived spontaneously after a year and delivered a healthy baby at term by caesarean section. She was followed up for the next two years, and the period was uneventful.

## Discussion

The diagnosis of cervical fibroid is typically made using ultrasonography, MRI, CECT, or a combination of ultrasonography with MRI or CT [8]. In our cases, the diagnosis was established by integrating ultrasonography with MRI or CT. Cervical fibroids are rare; thus, their management can be challenging. The choice of treatment is crucial for preserving fertility in women of reproductive age. To improve reproductive outcomes and address bleeding episodes, surgical intervention is often necessary.

Cervical fibroids present a surgical challenge for the gynaecologist due to their proximity to vital structures such as the ureters, bladder, rectum, and uterine arteries [8]. The ballooning of the cervix due to cervical fibroids alters the anatomical relations of these structures, increasing their risk of injury. A proper preoperative workup, a thorough knowledge of anatomy, and careful dissection of the bladder and ureters are required, as the anatomy is often distorted. Cervical fibroids are related to the bladder anteriorly, bilateral ureters laterally, and rectum posteriorly, making surgery quite difficult.

The treatment of choice for cervical fibroids is myomectomy or hysterectomy [7]. Myomectomy is the procedure of choice for cervical fibroids in women who desire to preserve their fertility [7]. A small prolapsing fibroid with a stalk protruding from the cervix can be removed via hysteroscopy using hysteroscopic scissors, a resectoscope, or a bipolar electrode [9]. Hysteroscopy allows for visual inspection of other cervical and endometrial polyps [9]. However, for large intracervical fibroids, management through open, laparoscopic, and even newer robotic approaches is challenging. There are no guidelines on the optimum surgical route, and treatment is tailored based on the patient's age, desire for fertility preservation, and the number, location, and size of the fibroids.

Laparotomic myomectomy has been described in a few earlier case reports [10]. However, the narrow operative field, even in open laparotomy, as well as the proximity to the ureters and bladder, make the procedure difficult. Preoperative ureteric catheterization is performed in most cases to delineate the altered course of the ureters and avoid injury. The increased postoperative morbidity due to abdominal scar and the higher risk of adhesion formation are other disadvantages of laparotomic myomectomy. Though laparoscopic myomectomy for large cervical fibroids has been attempted, the use of power morcellator has recently been limited by the US Food and Drug Administration (FDA), due to their risk of upstaging unsuspected uterine sarcomas, to only appropriately selected women, that too using a tissue containment system [8].

A robotic approach to cervical myomectomy has recently been described, though it remains an abdominal approach [11]. The vaginal approach for uterine leiomyoma excision using colpotomy has also been documented [12]. Liu et al. described vaginal natural orifice transluminal endoscopic surgery (vNOTES) for myomectomy, where a 6 cm uterine leiomyoma was resected transvaginally through an anterior colpotomy [13]. However, these myomectomies were done for uterine rather than cervical fibroids.

While medical options do not provide permanent treatment for fibroids, the use of gonadotropin-releasing hormone (GnRH) analogues preoperatively can effectively reduce fibroid size and intraoperative bleeding [14]. Utilizing GnRH analogues for three to four months before fibroid surgery reduces both uterine volume and fibroid size [14]. However, in our resource-constrained setting, GnRH analogues were not used for any of our patients.

In our institution, over five years, we have operated on a series of women with large intracervical fibroids, some extending to the body of the uterus or laterally to the broad ligament. All the women were younger than 40 years, where the incidence of leiomyosarcoma is lower, and the patients desired to preserve their uterus. The intraoperative injection of diluted vasopressin into the fibroid can reduce blood loss [15]. The injection of vasopressin into the fibroid capsule intraoperatively serves as an effective haemostatic measure, especially since tourniquet application is not possible [15]. We used adrenaline in a 1:200,000 dilution, which was injected into the fibroid capsule for haemostasis, as vasopressin was not available due to resource constraints. Bilateral uterine artery embolization at its origin from the internal iliac artery has been employed in certain cases to reduce blood loss [16]. However, its use in women desiring future childbirth remains controversial. Moreover, being a low-resource setting, the facility was not available at our institution. The shelling out of the myoma was done following Bonney’s principles diligently; that is, a transverse incision was made along its prominent proximal portion to ascertain the plane of cleavage between the tumour and its capsule. The most challenging aspect of cervical myomectomy is suturing the base after enucleation. Traction on the leiomyoma with the myoma screw helped us reach the base of the fibroid, which was then clamped. In our case, prophylactic double-J (DJ) stenting was not required as the risk of ureteric injury was negligible with the vaginal approach, since the entire process was intracapsular. 

Overall, the experience was quite satisfying, as all cases were approached with the understanding that due to the difficulty of accessing such lesions both vaginally and abdominally, we were skeptical about the outcomes and complications. However, all myomectomy cases were completed vaginally without any complications.

The drawback of this study is that it necessitated the expertise of an experienced vaginal surgeon. Additionally, cervical fibroids that were not palpable vaginally could not be removed.

## Conclusions

The management of cervical fibroids is challenging, and there is no consensus on the best surgical approach. A narrow operative field, proximity to the ureters, urinary bladder, and rectum, and consequently their risk of injury, and the risk of haemorrhage due to uterine artery injury, complicates the surgery even with an open laparotomy approach. Our case series demonstrates that vaginal myomectomy of a large intracervical fibroid, when performed by skilled hands, is safe with minimal risk of injury to adjacent organs as the dissection is performed within the capsule of the fibroid. This approach is ideal for women desiring fertility. Unlike open myomectomy, it requires no skin incision and has a minimal risk of postoperative adhesions. The only drawback is that it can only be used when the fibroid is palpable through the cervical lips.

## References

[1] Yang Q, Ciebiera M, Bariani MV, Ali M, Elkafas H, Boyer TG, Al-Hendy A (2022). Comprehensive review of uterine fibroids: developmental origin, pathogenesis, and treatment. Endocr Rev.

[2] Tiltman AJ (1998). Leiomyomas of the uterine cervix: a study of frequency. Int J Gynecol Pathol.

[3] Berek JS (2019). Berek and Novak's Gynecology. https://books.google.co.in/books/about/Berek_Novak_s_Gynecology.html?id=YJs1swEACAAJ&redir_esc=y.

[4] Takeuchi H, Kitade M, Kikuchi I (2006). A new enucleation method for cervical myoma via laparoscopy. J Minim Invasive Gynecol.

[5] Laganà AS, Vergara D, Favilli A (2017). Epigenetic and genetic landscape of uterine leiomyomas: a current view over a common gynecological disease. Arch Gynecol Obstet.

[6] Pavone D, Clemenza S, Sorbi F, Fambrini M, Petraglia F (2018). Epidemiology and risk factors of uterine fibroids. Best Pract Res Clin Obstet Gynaecol.

[7] Lethaby A, Vollenhoven B, Sowter M (2002). Efficacy of pre-operative gonadotrophin hormone releasing analogues for women with uterine fibroids undergoing hysterectomy or myomectomy: a systematic review. BJOG.

[8] Mujalda A, Kaur T, Jindal D, Sindhu V, Jindal P, Mujalda J (2023). Giant cervical fibroid: a surgical challenge. Cureus.

[9] Stamatellos I, Stamatopoulos P, Bontis J (2007). The role of hysteroscopy in the current management of the cervical polyps. Arch Gynecol Obstet.

[10] Abu Hashim H, Al Khiary M, El Rakhawy M (2020). Laparotomic myomectomy for a huge cervical myoma in a young nulligravida woman: a case report and review of the literature. Int J Reprod Biomed.

[11] Dou L, Zhang Y (2022). Tips and details for successful robotic myomectomy: single-center experience with the first 125 cases. J Clin Med.

[12] Davies A, Hart R, Magos AL (1999). The excision of uterine fibroids by vaginal myomectomy: a prospective study. Fertil Steril.

[13] Liu J, Lin Q, Blazek K, Liang B, Guan X (2018). Transvaginal natural orifice transluminal endoscopic surgery myomectomy: a novel route for uterine Myoma removal. J Minim Invasive Gynecol.

[14] Lethaby A, Vollenhoven B, Sowter M (2001). Pre-operative GnRH analogue therapy before hysterectomy or myomectomy for uterine fibroids. Cochrane Database Syst Rev.

[15] Shimanuki H, Takeuchi H, Kitade M, Kikuchi I, Kumakiri J, Kinoshita K (2006). The effect of vasopressin on local and general circulation during laparoscopic surgery. J Minim Invasive Gynecol.

[16] Mahajan NN, Soni RN, Mahajan KN (2007). Uterine artery ligation via the laparoscope prior to myomectomy. Fertil Steril.

